# Adequate target volume in total-body irradiation by intensity-modulated radiation therapy using helical tomotherapy: a simulation study

**DOI:** 10.1093/jrr/rrw115

**Published:** 2016-12-14

**Authors:** Ryosuke Takenaka, Akihiro Haga, Hideomi Yamashita, Keiichi Nakagawa

**Affiliations:** 1 Department of Radiology, University of Tokyo Hospital, 7-3-1, Hongo, Bunkyo-ku, Tokyo, 113-8655, Japan

**Keywords:** total-body irradiation, intensity-modulated radiation therapy, helical tomotherapy, set-up error, PTV margin

## Abstract

Recently, intensity-modulated radiation therapy (IMRT) has been used for total-body irradiation (TBI). Since the planning target volume (PTV) for TBI includes the surrounding air, a dose prescription to the PTV provides high fluence to the body surface. Thus with just a small set-up error, the body might be exposed to a high-fluence beam. This study aims to assess which target volume should be prescribed the dose, such as a clinical target volume (CTV) with a margin, or a CTV that excludes the surface area of the skin. Three treatment plans were created for each patient: the 5-mm clipped plan (Plan A), the 0-mm margin plan (Plan B) and the 5-mm margin plan (Plan C). The CTV was the whole body. PTVs were the CTV with the exception of 5 mm from the skin surface in Plan A, equal to the CTV in Plan B, and the CTV with a 5 mm margin in Plan C. The prescribed dose was 12 Gy in six fractions. To assess the influence of the set-up error, dose distributions were simulated on computed tomography (CT) images shifted 2 pixels (= 4.296 mm), 5 pixels (= 10.74 mm) and 10 pixels (= 21.48 mm) in the lateral direction from the original CT. With a set-up error of 10.74 mm, V_110%_ was 8.8%, 11.1% and 23.3% in Plans A, B and C, respectively. The prescription to the PTV containing the surrounding air can be paradoxically vulnerable to a high-dose as a consequence of a small set-up error.

## INTRODUCTION

Total-body irradiation (TBI) has been performed as a conditioning regimen for allogeneic hematopoietic stem cell transplantation (HSCT). Although several randomized trials have suggested that HSCT with TBI achieved superior outcomes compared with those with non-TBI–containing regimens [1], TBI also provokes substantial toxicities in multiple organs, especially the lungs [2, 3]. Lung-shielding is adopted in many institutions, but the methods of shielding are heterogeneous, and no one knows which method is superior to the others [4]. Recently, intensity-modulated radiation therapy (IMRT) using helical tomotherapy to shield the organs at risk has been shown to enable delivery of TBI in a more secure way [5–7].

In inverse planning of IMRT, a high fluence is administered to tangential beam segments near the skin in order to counter the build-up region [8]. This is the reason for increasing the skin dose when the clinical target volume (CTV) is near the skin, such as in TBI. In many other treatment areas, for example, in breast or head-and-neck cancers, it is recommended that the skin is excluded from the target volume [9]. Moreover, because the CTV comprises the whole body in TBI, the planning target volume (PTV) contains the surrounding air, and the dose prescription to the PTV provides a much higher fluence to the body surface. Thus, the body might be exposed to a high-fluence beam as a consequence of a small set-up error. This logic generates a hypothesis that a plan with a PTV margin can be paradoxically vulnerable to set-up errors in TBI by IMRT. The purpose of this study was to assess which target volume is acceptable—a CTV with a margin or a CTV that excludes the surface area of the skin—and how much set-up error is acceptable.

## MATERIALS AND METHODS

### Patients

A total of 10 patients underwent hematopoietic stem cell transplantations with myeloablative preparation regimens and received TBI by IMRT, using helical tomotherapy. The present study was ethically approved by the institutional review board of The University of Tokyo Hospital (Reference number 3372). Written informed consent was obtained from all patients.

Patients were immobilized in a supine position using Vac-Lok cushions and thermoplastic masks for the head. Planning computed tomography (CT) images were acquired with 5- mm slice thickness from a 16-slice CT system (Aquilion LB, Toshiba Medical Systems). The field of view (FOV) was 55 cm × 55 cm.

### Planning

**Fig. 1.**
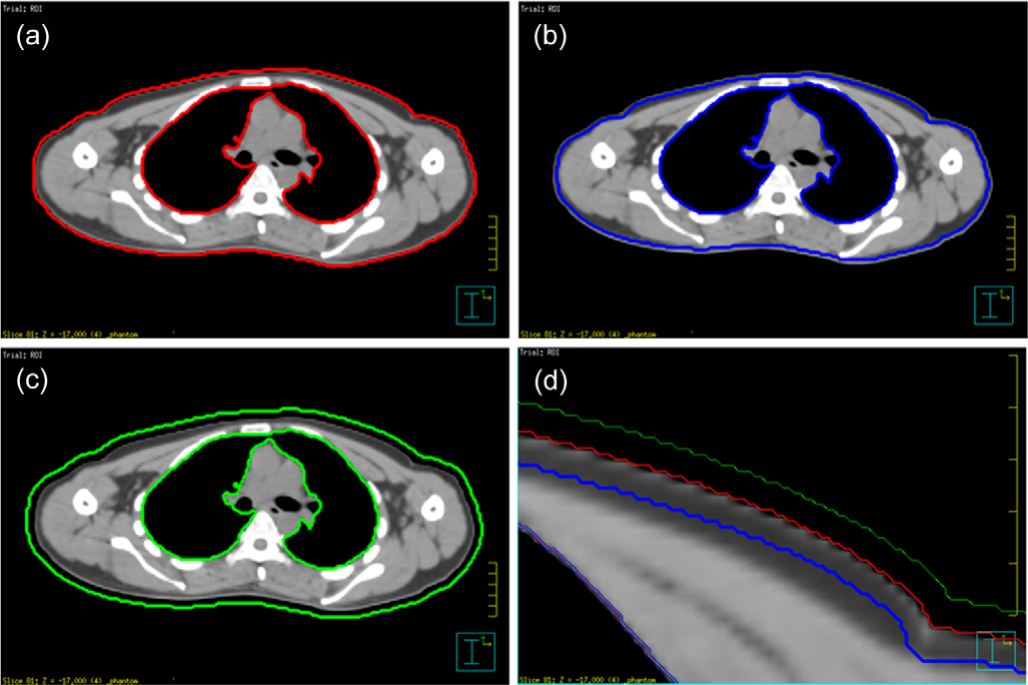
The delineations of target volumes. (a) CTV includes the whole body except for the lungs and this is equal to the PTV_0_. (b) The PTV_–_ is reduced from the CTV by subtracting the 5 mm of the skin surface. (c) The PTV_+_ is the CTV with a 5 mm margin. (d) An enlargement of an area near the skin surface.

Three treatment plans were created for 10 patients undergoing TBI: Plan A (the 5-mm clipped plan), Plan B (the 0-mm margin plan) and Plan C (the 5-mm margin plan). The target volumes were as follows (Fig. 1):

- CTV including the whole body except for the lungs
- Plan A: PTV (PTV_–_) was the CTV minus the 5 mm under the skin surface.
- Plan B: PTV (PTV_0_) was equal to the CTV.
- Plan C: PTV (PTV_+_) was the CTV plus a 5 mm margin.

A total of 12 Gy in six fractions was prescribed to 95% of the PTV_–_ volume in Plan A. In the other two plans, the prescribed volumes of the PTV (PTV_0_ or PTV_+_) were adjusted so that 12 Gy was irradiated to 95% of the PTV.

In terms of normal tissue constraints, V_10 Gy_ (the percentage of the volume receiving 10 Gy or more) of the lung was suppressed to <10%, and the maximum dose to the kidney was suppressed to <12 Gy.

Optimization was performed by the TomoTherapy Planning Station (TomoHDA System ver. 2.0.2, Hi-Art ver. 4). The field width was 5.0 cm and the pitch was 0.43. The modulation factor was 2.5 in most of the patients, but decreased to 2.0 in smaller patients e.g. children. A fixed-jaw mode was adopted.

### Simulation of set-up error

In the TomoTherapy Planning Station, a dose grid of CT was reconstructed into 256 × 256 × *N* pixels (*N* is the number of planning CT slices). As the FOV of the planning CT is 55 × 55 cm, 1 pixel equals 0.2148 cm ( = 55/256 cm) in an axial slice.

To assess the influence of the set-up error on the delivered dose, original CT images and contours were re-imported into the TomoTherapy Planning Station as phantoms. These secondary CT images and contours were shifted 2 pixels ( = 4.296 mm), 5 pixels ( = 10.74 mm) and 10 pixels ( = 21.48 mm) in the lateral direction from the original CT datasets. Then, the dose distributions of each plan were simulated on shifted CT images in delivery quality assurance (DQA) mode.

To assess CTV coverage, the V_90%_ (volume receiving 90% of the prescription dose), V_100%_ and D_98%_ (dose exceeded in 98% of the volume) of the CTV were evaluated in the three original plans and each shifted plan. To assess the high-dose areas, the V_110%_ and D_2%_ of the CTV were evaluated. Moreover, to focus on the skin dose, the D_2%_ and D_98%_ of the area within 5 mm of the body surface were calculated.

Because the lung was an organ at risk, V_10 Gy_, V_12 Gy_ and the mean dose to the lung were evaluated.

All patients’ body heights were >128 cm, the upper limit of radiation target length by helical tomotherapy, so the actual plans were divided into the top of skull to middle femurs and from middle femurs to toes. In the present simulation, the plans were only performed from the top of skull to middle femurs.

Statistical analysis was performed using JMP, version 11.2 (SAS Institute Inc.). Dose–volume parameters of the original plans and shifted plans were compared using a paired Student's *t* test, and the differences are reported as statistically significant at *P* < 0.05 (two-tailed).

## RESULTS

### Dose–volume parameters in original plans

Patient characteristics are shown in Table 1.

**Table 1.** Patient characteristics

Pt	Age (years)	Gender	Height (cm)	Weight (kg)	BMI	Disease
1.	55	M	166	50	18.0	AML
2.	11	F	135	27	14.6	T-ALL
3.	31	M	173	75	24.9	CML
4.	43	F	159	62	24.6	MDS
5.	45	M	176	74	23.7	T-ALL
6.	43	F	168	52	18.5	B-ALL
7.	37	M	159	47	18.6	ATL
8.	42	F	163	50	18.7	AML
9.	43	M	176	58	18.9	AML
10.	41	F	157	47	19.0	B-ALL

BMI = body mass index (calculated as weight in kilograms divided by the square of height in meters), AML = acute myeloid leukemia, T-ALL = T-cell acute lymphoblastic leukemia, CML = chronic myeloid leukemia, MDS = myelodysplastic syndrome, B-ALL = B-cell acute lymphoblastic leukemia, ATL = adult T-cell leukemia.

In order to adjust D_95%_ of PTV_–_ to 12 Gy, the mean prescribed volume of the PTV was set at 94.6% (from 93.0% to 95.0%) of PTV_0_ in Plan B, and 93.1% (from 91.0% to 94.0%) of PTV_+_ in Plan C.

In the original plans, V_100%_ of the CTV [average ± standard deviation (SD)] was 93.3 ± 0.6%, 94.9 ± 0.4% and 95.5 ± 0.3% in Plans A, B and C, respectively. Other dose–volume parameters are shown in Table 2. V_90%_ and V_100%_ of the CTV were lower in Plan A than in the other plans (*P* < 0.001). On the other hand, V_110%_ was higher in Plan C than in the other plans (*P* < 0.001). Because V_100%_ of PTV_–_ (which was the CTV except for the body surface) was adjusted as 95% in the three plans, the CTV dose differences between the three plans depended mainly on the dose to the area within 5 mm of the body surface. The mean doses to the area within 5 mm of the body surface were 12.4 ± 0.2 Gy, 12.7 ± 0.2 Gy and 13.1 ± 0.1 Gy in Plans A, B and C, respectively.

**Table 2.** Dose–volume parameters of the original plans and the shifted plans

		Plan A (5-mm clipped plan)				Plan B (0-mm margin plan)				Plan C (5-mm margin plan)			
	Average ± SD	Original	2 pixels	5 pixels	10 pixels	Original	2 pixels	5 pixels	10 pixels	Original	2 pixels	5 pixels	10 pixels
CTV	V_90%_ (%)	97.9 ± 0.2	97.7 ± 0.2	96.0 ± 0.7	92.4 ± 1.0	98.6 ± 0.3	98.4 ± 0.2	97.6 ± 0.3	95.5 ± 0.6	98.7 ± 0.2	98.6 ± 0.2	97.9 ± 0.3	96.6 ± 0.6
	V_100%_ (%)	93.3 ± 0.6	93.0 ± 0.7	90.8 ± 1.6	84.6 ± 4.4	94.9 ± 0.4	94.8 ± 0.5	93.7 ± 0.9	90.8 ± 1.2	95.5 ± 0.3	95.5 ± 0.4	94.8 ± 0.4	93.0 ± 0.7
	V_110%_ (%)	2.0 ± 1.0	5.6 ± 8.0	8.8 ± 10.5	10.7 ± 7.5	5.8 ± 6.8	8.2 ± 7.1	11.1 ± 7.2	15.9 ± 8.3	18.1 ± 7.1	20.5 ± 8.1	23.3 ± 7.0	28.5 ± 6.1
	D_2%_ (Gy)	13.2 ± 0.1	13.3 ± 0.1	13.4 ± 0.1	13.4 ± 0.2	13.3 ± 0.2	13.5 ± 0.2	14.0 ± 0.3	14.6 ± 0.3	13.8 ± 0.2	14.0 ± 0.1	14.9 ± 0.3	16.2 ± 0.6
	D_98%_ (Gy)	10.8 ± 0.1	10.7 ± 0.1	9.8 ± 0.3	8.8 ± 0.1	11.1 ± 0.2	11.0 ± 0.1	10.5 ± 0.2	9.5 ± 0.3	11.2 ± 0.1	11.2 ± 0.1	10.7 ± 0.2	9.8 ± 0.3
Lung	Mean dose (Gy)	9.4 ± 0.1	9.5 ± 0.2	9.8 ± 0.1	10.3 ± 0.2	9.3 ± 0.2	9.4 ± 0.2	9.7 ± 0.2	10.3 ± 0.2	9.4 ± 0.1	9.5 ± 0.2	9.8 ± 0.2	10.5 ± 0.2
	V_10 Gy_ (%)	5.3 ± 3.6	12.0 ± 3.9	25.1 ± 3.7	41.6 ± 3.1	6.4 ± 5.3	12.2 ± 4.4	23.9 ± 3.8	41.8 ± 3.4	7.0 ± 2.8	13.5 ± 2.9	26.1 ± 2.6	43.2 ± 3.0
	V_12 Gy_ (%)	0.0 ± 0.0	0.1 ± 0.1	4.9 ± 1.2	21.9 ± 1.5	0.0 ± 0.0	0.1 ± 0.1	4.6 ± 1.9	21.5 ± 1.9	0.0 ± 0.0	0.1 ± 0.1	5.1 ± 1.2	21.8 ± 2.4
Within 5 mm of the body surface	Mean dose (Gy)	12.4 ± 0.2	12.4 ± 0.2	12.1 ± 0.3	11.8 ± 0.2	12.7 ± 0.2	12.8 ± 0.2	12.8 ± 0.2	12.6 ± 0.1	13.1 ± 0.1	13.2 ± 0.1	13.4 ± 0.1	13.3 ± 0.1
	D_2%_ (Gy)	13.3 ± 0.1	13.4 ± 0.1	13.5 ± 0.2	13.5 ± 0.2	13.5 ± 0.2	14.1 ± 0.2	15.1 ± 0.3	15.1 ± 0.3	14.3 ± 0.3	15.0 ± 0.4	16.9 ± 0.9	17.8 ± 1.2
	D_98%_ (Gy)	9.8 ± 0.4	9.7 ± 0.4	8.4 ± 1.0	6.8 ± 0.3	11.1 ± 0.5	11.0 ± 0.5	10.4 ± 0.7	8.9 ± 0.3	12.0 ± 0.4	11.9 ± 0.4	11.6 ± 0.4	10.8 ± 0.5

SD = standard deviation, CTV = clinical target volume.

The lung was well shielded; V_12 Gy_ of the lung was ~0% in all of the plans. V_10 Gy_ was 5.3 ± 3.6%, 6.4 ± 5.3% and 7.0 ± 2.8% in Plans A, B and C, respectively.

### Changes in dose–volume parameters in shifted plans

**Fig. 2.**
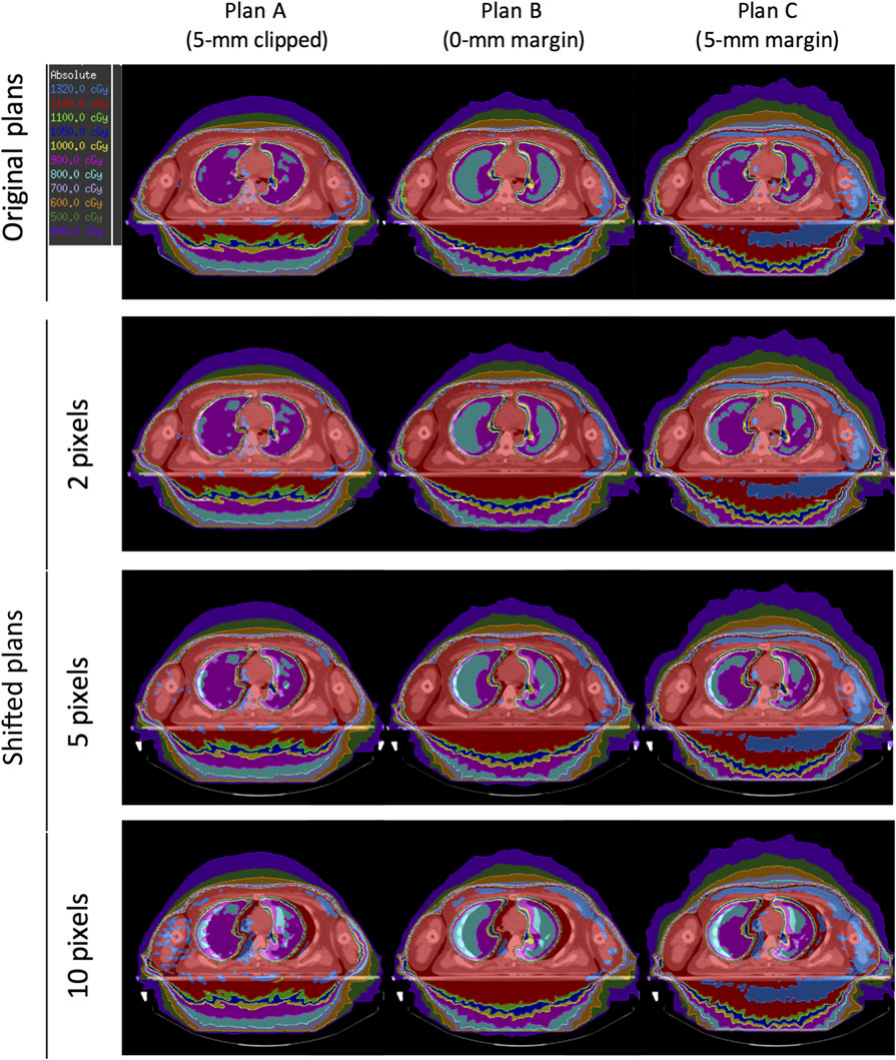
Examples of dose distributions. In Plan C, the area just under the skin surface tends to receive a high irradiation dose. When CT images are shifted to the left side, the body is exposed to a high-fluence beam and the high-dose area is enlarged.

**Fig. 3.**
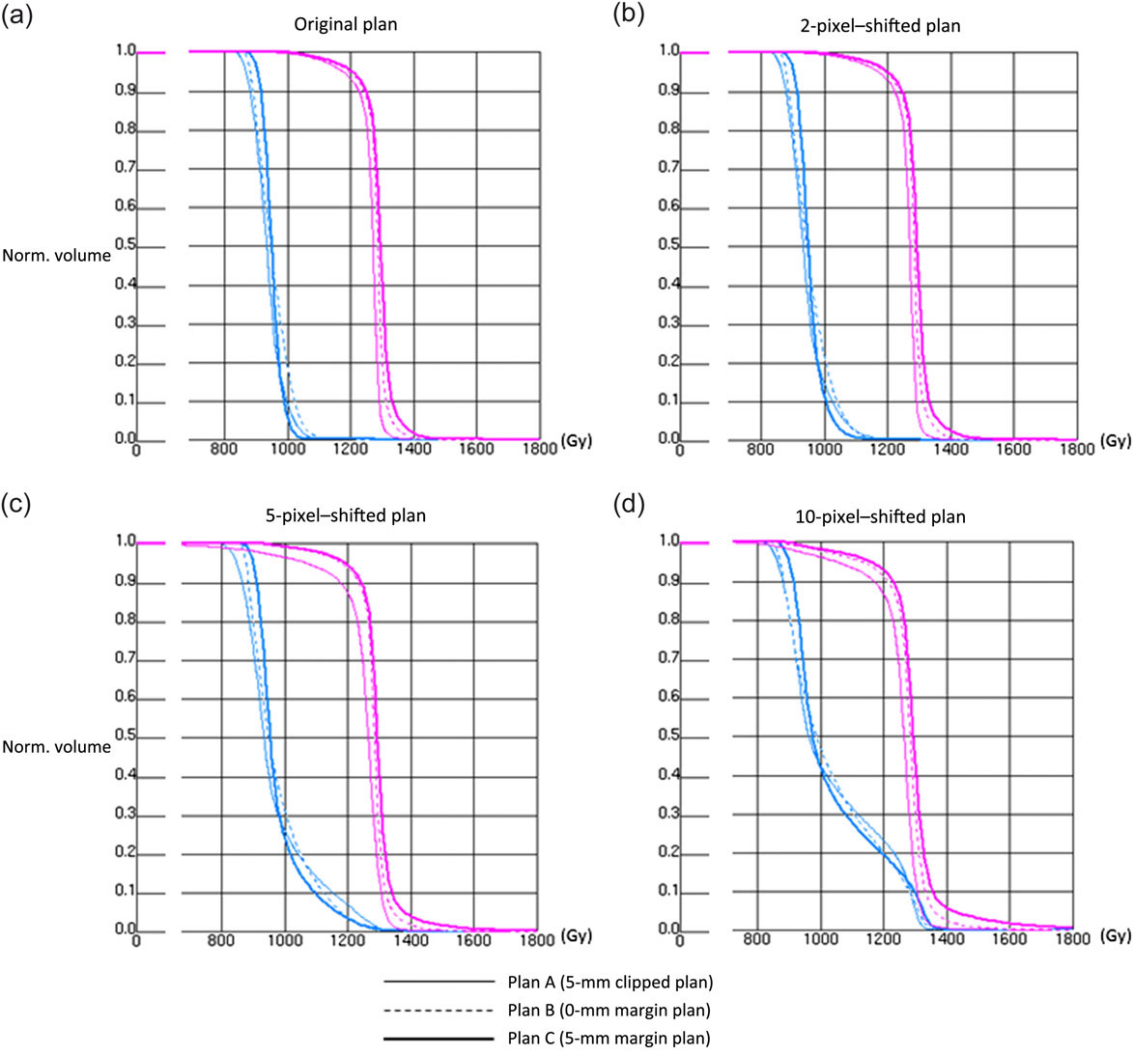
Examples of dose–volume histograms of CTVs and lungs for the original plans (a) and shifted plans (b, c, d). The thin solid lines show the 5-mm clipped plan, the thin dashed lines show the 0-mm margin plan, and the thick solid lines show the 5-mm margin plan. The larger the amount of shift, the worse the homogeneity.

Figure 2 shows examples of dose distributions, and Fig. 3 shows examples of dose–volume histograms in the original plans and shifted plans. Similar results were obtained for other patients.

When CT images were shifted in the lateral direction, both the high-dose (>110% of the prescribed dose irradiated) and low-dose areas (<90% of the prescribed dose irradiated) were enlarged. As a result, V_100%_ of the CTV was decreased with the increasing shift distance in all three plans (Table 2), but an image shift of 2 pixels (4.3 mm) had little influence on dose distribution in either plan. The differences in V_100%_ between the original plans and the 2-pixel–shifted plans were not statistically significant (*P* = 0.07 in Plan A, *P* = 0.15 in Plan B, and *P* = 0.72 in Plan C). When images were shifted by 5 pixels, V_100%_ of the CTV was decreased significantly (*P* < 0.001 in both plans). The decrease in V_100%_ of the CTV by image shift was largest in Plan A. In this plan, V_100%_ of the CTV decreased to <90% when images were shifted 10 pixels (21.5 mm), while it was preserved by >90% in the other two plans. On the other hand, V_110%_ of the CTV was highest in Plan C; and D_2%_ reached 14.9 Gy when the images were shifted 5 pixels (10.7 mm).

The lung dose was equal in the three plans. V_10 Gy_ of the lung was suppressed to 12% and 25% when the images were shifted 2 and 5 pixels (4.3 mm and 10.7 mm), respectively, in either plan. However, V_10 Gy_ of the lung reached 40% when images were shifted 10 pixels (21.5 mm).

## DISCUSSION

The dose–volume histogram parameters of the original plans in the present study are similar to those in past reports about TBI by IMRT [5, 6]. With the shift of the CT image simulating set-up error in the lateral direction, a high-dose area emerged especially at the skin surface, and it was larger in Plan C (the 5-mm margin plan) than in the other two plans. In contrast, the low-dose area was larger in Plan A (the 5-mm clipped plan). We used V_110%_ and V_90%_ as indicators of high- and low-dose areas, respectively, according to the guidelines published by the American College of Radiology and the American Society for Radiation Oncology [10]. Their guidelines recommended maintaining dose inhomogeneity within ±10%. We considered it was not permissible that V_110%_ of the CTV exceeded 10% or that V_90%_ fell below 90%. With respect to the lung dose, we considered it was not permissible that V_10 Gy_ exceeded 40% [11]. A set-up error of 2 pixels (4.3 mm) influenced dose distribution within the allowable range in Plans A and B. The V_110%_ of Plan C was somewhat high, even in the original plan. This is partially because 12 Gy was irradiated to 95% of the PTV. It proved impossible to meet the dose constraint in which D_95%_ of the PTV was 12 Gy and V_110%_ of PTV was <10% if the PTV contained air. Lower dose prescription made it possible to decrease the V110% of the PTV. Actually, the dose prescription in some of the other studies was lower (D_50%_ of PTV was 12 Gy) [7, 12], but the prescription of D_95%_ is also popular [5, 6], and dose–volume histogram parameters of the original plans in the present study are similar in such past reports.

To the best of our knowledge, to date there have not been any reports published that assess the influence of set-up errors on the dose distribution for TBI by IMRT using helical tomotherapy. We simulated dose distribution in 10 patients and obtained reproducible results. Mancosu *et al*. [13] reported that in total marrow irradiation (TMI) by volumetric-modulated arc therapy (VMAT), the mean dose to the body differed from the original plan by <0.1% with a set-up error of 5 mm in the lateral direction. Although the high-dose area on the body surface, due to build-up effects, is probably larger in TBI than in TMI, the CTV extends closer to the skin surface in TBI, so our results are compatible with those in the past report in terms of tolerating a set-up error of 5 mm.

It depends on the policy of each facility what target volume should be adopted in response to the results of the present study. Some investigators may judge a skin dose of ≤20 Gy as acceptable and adopt the 5-mm margin plan. However, it is somewhat nonsensical to set a PTV margin containing air because the area protruding out of the body contour on the planning CT is exposed to a high dose, even in the 0-mm margin plan. Others may dislike the appearance of an unpredictable high-dose area. Actually, the skin dose even in the 5-mm clipped plan is much higher than in conventional long source-to-surface distance technique or the moving-couch technique [14].

It is an important finding that the dose distribution of an IMRT plan in which the target contains surrounding air with a large PTV margin, can be paradoxically vulnerable to the appearance of a high-dose area due to a set-up error. One should not get comfortable with setting a large PTV margin, but should instead strictly immobilize patients.

The present study has some limitations. This is a simulation study of a treatment planning system. Therefore, it is controversial whether the dose distribution has been accurately calculated in build-up regions. Nonetheless, this simulation reflected the tendencies of three plans with different target volumes. It would be helpful (for estimating the skin dose) to put fluoroglass dosimeters on the skin surface of patients.

The simulation of set-up error in this study is only related to the homogeneous lateral shift. We also simulated a homogeneous shift of 10 mm in other directions (anterior and superior). The skin surface protruded out of the body contour of the planning CT was exposed to a high dose, and this effect was significant in the 5-mm margin plan (see supplementary Table 1). This result conformed to that of the lateral movement. Other set-up errors (contortion; the position of arms, legs or jaw; gains in weight from the planning CT to irradiation; etc.) can't be assessed. The evaluation of the influence of such complicated errors on dose distribution is outside the reach of this study and would be a challenging research project. Although the body didn't protrude from the body contour of the planning CT by more than 5 mm, at least the time of taking megavoltage CT for the registration in our experience, to evaluate whether the body contour protrude in larger quantity during the actual beam-on-time is a future issue. Reconstructing the position of the whole body using the delivery sinogram of helical tomotherapy during the actual treatment may make it possible to calculate the actual delivered dose [15].

In conclusion, the dose distribution of the plan in which the target contains surrounding air can be paradoxically vulnerable to appearance of a high-dose area, especially at the skin surface, due to a set-up error. A set-up error of ~5 mm influences dose distribution within the allowable range for TBI by IMRT.

## Supplementary Material

Supplementary Data
