# Supplementary material for: Adequate target volume in total-body irradiation by intensity-modulated radiation therapy using helical tomotherapy: a simulation study
Source: J Radiat Res. 2016 Dec 14;58(2):210–6. doi: 10.1093/jrr/rrw115 (PMC5439372; doi:10.1093/jrr/rrw115)
Supplement: Supplementary Data [file imrt-tbi_manuscript_jrr_supplementarytable.docx]

Supplementary Table. Dose-volume parameters of the original plans and the shifted plans simulating 3D-movement.

|  |  | The plan A (the 5-mm clipped plan) | | | |  | The plan B (the 0-mm margin plan) | | | |  | The plan C (the 5-mm margin plan) | | | |
| --- | --- | --- | --- | --- | --- | --- | --- | --- | --- | --- | --- | --- | --- | --- | --- |
|  | Average ± SD | Original | Anterior  10.74 mm | Lateral  10.74 mm | Superior  10 mm |  | Original | Anterior  10.74 mm | Lateral  10.74 mm | Superior  10 mm |  | Original | Anterior  10.74 mm | Lateral  10.74 mm | Superior  10 mm |
| CTV | V_90%_ (%) | 97.9 ± 0.2 | 96.0 ± 0.6 | 96.0 ± 0.7 | 97.8 ± 0.2 |  | 98.6 ± 0.3 | 97.5 ± 0.3 | 97.6 ± 0.3 | 98.5 ± 0.4 |  | 98.7 ± 0.2 | 97.8 ± 0.4 | 97.9 ± 0.3 | 98.7 ± 0.2 |
|  | V_100%_ (%) | 93.3 ± 0.6 | 91.4 ± 1.2 | 90.8 ± 1.6 | 93.2 ± 0.6 |  | 94.9 ± 0.4 | 94.7 ± 0.4 | 93.7 ± 0.9 | 94.8 ± 0.5 |  | 95.5 ± 0.3 | 95.6 ± 0.4 | 94.8 ± 0.4 | 95.6 ± 0.5 |
|  | V_110%_ (%) | 2.0 ± 1.0 | 9.6 ± 6.4 | 8.8 ± 10.5 | 5.3 ± 8.4 |  | 5.8 ± 6.8 | 19.0 ± 10.2 | 11.1 ± 7.2 | 6.8 ± 8.0 |  | 18.1 ± 7.1 | 40.1 ± 9.6 | 23.3 ± 7.0 | 19.2 ± 7.5 |
|  | D_2%_ (Gy) | 13.2 ± 0.1 | 13.5 ± 0.1 | 13.4 ± 0.1 | 13.2 ± 0.1 |  | 13.3 ± 0.2 | 14.8 ± 0.4 | 14.0 ± 0.3 | 13.3 ± 0.2 |  | 13.8 ± 0.2 | 16.1 ± 0.7 | 14.9 ± 0.3 | 13.8 ± 0.2 |
|  | D_98%_ (Gy) | 10.8 ± 0.1 | 9.8 ± 0.2 | 9.8 ± 0.3 | 10.7 ± 0.1 |  | 11.1 ± 0.2 | 10.4 ± 0.3 | 10.5 ± 0.2 | 11.0 ± 0.2 |  | 11.2 ± 0.1 | 10.7 ± 0.3 | 10.7 ± 0.2 | 11.2 ± 0.1 |
| Lung | Mean dose (Gy) | 9.4 ± 0.1 | 9.8 ± 0.1 | 9.8 ± 0.1 | 9.4 ± 0.2 |  | 9.3 ± 0.2 | 9.7 ± 0.2 | 9.7 ± 0.2 | 9.4 ± 0.2 |  | 9.4 ± 0.1 | 9.9 ± 0.2 | 9.8 ± 0.2 | 9.5 ± 0.2 |
|  | V_10Gy_ (%) | 5.3 ± 3.6 | 26.9 ± 3.1 | 25.1 ± 3.7 | 6.0 ± 3.5 |  | 6.4 ± 5.3 | 27.1 ± 2.7 | 23.9 ± 3.8 | 6.4 ± 4.9 |  | 7.0 ± 2.8 | 30.3 ± 4.5 | 26.1 ± 2.6 | 7.5 ± 3.6 |
|  | V_12Gy_ (%) | 0.0 ± 0.0 | 3.9 ± 1.4 | 4.9 ± 1.2 | 0.0 ± 0.0 |  | 0.0 ± 0.0 | 4.1 ± 1.8 | 4.6 ± 1.9 | 0.0 ± 0.0 |  | 0.0 ± 0.0 | 4.6 ± 2.2 | 5.1 ± 1.2 | 0.0 ± 0.0 |
| Within 5 mm from the body surface | Mean dose (Gy) | 12.4 ± 0.2 | 12.2 ± 0.2 | 12.1 ± 0.3 | 12.3 ± 0.2 |  | 12.7 ± 0.2 | 13.2 ± 0.2 | 12.8 ± 0.2 | 12.7 ± 0.2 |  | 13.1 ± 0.1 | 14.1 ± 0.2 | 13.4 ± 0.1 | 13.1 ± 0.1 |
|  | D_2%_ (Gy) | 13.3 ± 0.1 | 13.6 ± 0.1 | 13.5 ± 0.2 | 13.3 ± 0.2 |  | 13.5 ± 0.2 | 15.4 ± 0.3 | 15.1 ± 0.3 | 13.6 ± 0.2 |  | 14.3 ± 0.3 | 17.7 ± 1.3 | 16.9 ± 0.9 | 14.4 ± 0.4 |
|  | D_98%_ (Gy) | 9.8 ± 0.4 | 8.3 ± 0.4 | 8.4 ± 1.0 | 9.4 ± 0.8 |  | 11.1 ± 0.5 | 10.7 ± 0.6 | 10.4 ± 0.7 | 10.7 ± 0.8 |  | 12.0 ± 0.4 | 12.2 ± 0.4 | 11.6 ± 0.4 | 11.8 ± 0.6 |

Abbreviations: SD, standard deviation; CTV, clinical target volume.
